# Spondin-2 (SPON2), a More Prostate-Cancer-Specific Diagnostic Biomarker

**DOI:** 10.1371/journal.pone.0037225

**Published:** 2012-05-15

**Authors:** Xiaolong Qian, Changling Li, Bo Pang, Meng Xue, Jian Wang, Jianguang Zhou

**Affiliations:** 1 Department of Medical Molecular Biology, Beijing Institute of Biotechnology, Beijing, P. R. China; 2 Department of Breast Pathology and Research, State Key Lab of Breast cancer Research, Cancer Hospital of Tianjin Medical University, Tianjin, P. R. China; 3 Cancer Institute, Peking Union Medical College and Chinese Academy of Medical Science, Beijing, P. R. China; Ottawa Hospital Research Institute, Canada

## Abstract

**Background:**

Prostate-specific antigen (PSA) screening, although common, has recently been called into question. To find prostate cancer (PCa) diagnostic biomarkers that can make up for the defects of PSA, we compared the secretomes of several benign and PCa cell lines, selected candidate molecules, and then confirmed their clinical value.

**Methodology/Principal Findings:**

We first identified extracellular proteins by two-dimensional gel electrophoresis (2-DE) coupled with liquid chromatography-tandem mass spectrometry (LC-MS/MS) identification. We then validated the secreted proteins on a cellular level, and finally determined whether they could be used as PCa diagnostic biomarkers using prostate tissue and serum specimens of Chinese volunteers by immunohistostaining and sandwich ELISA. We obtained credible extracellular protein 2-DE graphs of prostate cell lines. The 5 spots that showed superior repeatability were selected for LC-MS/MS analysis, which identified seven candidate molecules. One of the candidate molecules, spondin-2 (SPON2), was only expressed in the conditioned media (CM) of androgen receptor (AR) positive PCa cell lines. Using tissue microarray by immunohistostaining, we found SPON2 to be over-expressed in PCa. SPON2 staining was more intense in Gleason score sum 7–8 and in PCa patients with metastasis. By receiver operator characteristic (ROC) curve analysis, we found that the serum SPON2 level was elevated in PCa patients, showing sensitivity and specificity suitable for diagnostic use. We also found that SPON2 could be used to identify PCa patients with serum PSA levels no higher than 10 ng/ml from healthy elderly men.

**Conclusion/Significance:**

SPON2 is a new serum and histological diagnostic biomarker for PCa. It can avoid some of the problems of PSA testing and was here found to offer relatively high sensitivity and specificity relative to PSA.

## Introduction

In the U.S., prostate cancer (PCa) alone accounts for 28% of all cancer cases in men [1]. Prostate-specific antigen (PSA), an androgen-regulated serine protease produced by both normal prostate epithelial cells and prostate cancer (PCa), is the most commonly used serum biomarker for PCa [2]. The determination of serum PSA levels and digital rectal examinations (DRE) are recommended by the majority of clinical guidelines for early detection of prostate cancer [3].

Although PSA screening has many advantages, it has been questioned in recent years. One of the major disadvantages of PSA is that it cannot be used to monitor healthy men for prostate cancer with simultaneous high sensitivity and high specificity [4]. The level of total PSA is elevated in acute prostatitis and benign prostatic hyperplasia (BPH) [5], [6]. Among men with slightly elevated PSA levels at 4–10 ng/ml, four men have to undergo biopsy to detect one man with PCa [3]. PSA screening may also cause misdiagnosis and prevent patients from seeking therapy in a timely manner. Thompson et al. discovered that among 2950 elderly men with a PSA level of 4.0 ng per milliliter or less, PCa was diagnosed in 449; 67 of them had a Gleason score sum of 7 or higher [7]. These examples highlight the urgency of discovering novel prostate-cancer-specific serum biomarkers that can discern life-threatening PCas, as well as PCas with serum PSA levels ≤10 ng/ml.

Here we compared the concentrated condition medium (CM) protein two-dimensional gel electrophoresis (2-DE) graph of BPH-1 (a BPH epithelial cell line, and its cytokeratin expression profile is consistent with a prostatic luminal epithelial cell [8]), LNCaP, and C4-2 (a derivative cell line of LNCaP that is androgen-independent, highly tumorigenic, and metastatic [9]). About 400 silver-staining-positive spots were obtained per 2-DE gel. After the elimination of background noise, standardization, and match analysis, we chose 5 spots whose differences appeared stable for LC-MS/MS identification. Seven candidate molecules were identified. One of the candidate molecules, SPON2, was only expressed in the CM of androgen-receptor-positive (AR–positive) PCa cell lines.

By tissue microarray immunohistochemical staining, we found that SPON2 expression in PCa tissues was higher than in normal prostate and BPH tissues. SPON2 expression was higher in PCa tissues with Gleason scores of 7–8 and in those from metastatic patients. Using a sandwich ELISA protocol, we compared 13 healthy, elderly men to 70 PCa patients and found that the serum SPON2 level was elevated in the latter. The area under the receiver operator characteristic (ROC) curve (AUC) of serum SPON2 was larger than 0.90, indicating a higher diagnostic value. The ROC curve also suggested that serum SPON2 concentrations of about 8 ng/mL gave the highest accuracy and best positive and negative predictive values–97.2% and 100%, respectively, as well as best sensitivity (100%) and specificity (84.6%). Comparing the ROC curves of serum SPON2 and PSA of PCa patients with PSA levels below 10 ng/mL to healthy controls, SPON2 was found to differentiate PCa patients with serum PSA≤10 ng/ml from healthy, elderly aged men, while PSA could not.

## Results

### Screening of candidate molecules in CM of PCa cell lines

**Figure 1:**
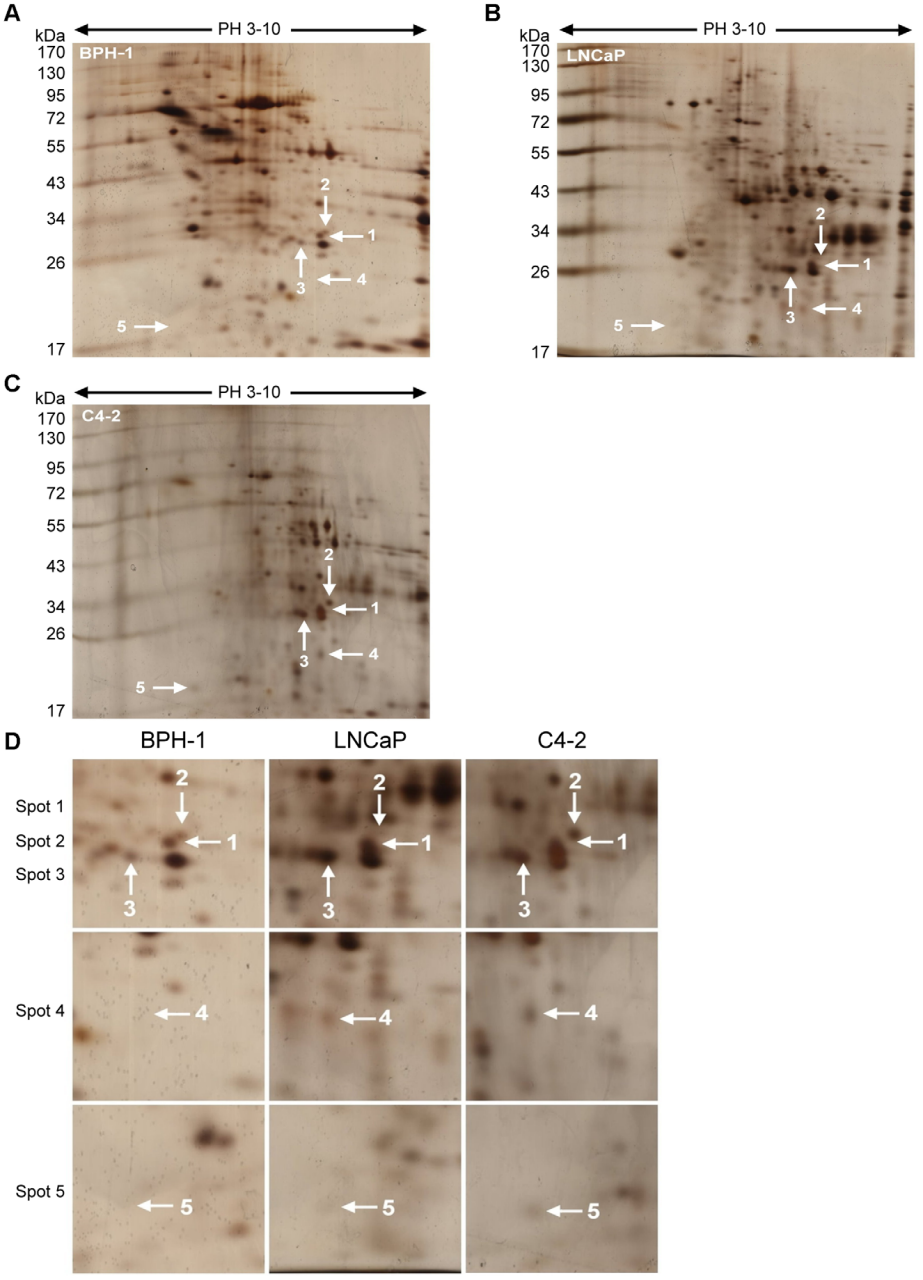
Two-dimensional gel electrophoresis profiles of condition media from BPH-1, LNCaP, and C4-2 cells. Two-dimensional electrophoregram of condition media from (A) BPH-1, (B) LNCaP, and (C) C4-2 cells are shown. (D) Enlarged corresponding areas of selected protein spots from these cell lines are also shown. Proteins in condition media of all cells were resolved by two-dimensional gel electrophoresis and silver-stained. White arrows indicate the differential spots.

Eighty micrograms of concentrated and purified extracellular proteins in CM of BPH-1, LNCaP, and C4-2 were separated by 2-DE. Scanned images of silver-stained 2-DE gels are shown in Figure 1A–C. The results were analyzed using PDQuest software, revealing about 400 protein spots per gel, of which five were up-regulated in CM from LNCaP and C4-2 cells in three independent repeated trials (The corresponding regions are enlarged and shown in Figure 1D.). The five differentially expressed protein spots were further detected by LC-MS/MS, and seven candidate molecules were identified: triosephosphate isomerase 1 (TPI1), thrombospondin 1 (THBS1), phosphoglycerate mutase 1 (PGAM1), spondin-2 (SPON2), syndecan binding protein 1 (ST1), peroxiredoxin 1 (PRDX1), and nucleophosmin (NPM1). As shown in Table 1, all seven proteins had high sequence coverage and Mascot scores. The probability-based Mowse score of each spot and matched peptides of each protein are shown in Figure S1. We were unable to determine which of the four candidate proteins identified in Spot 1 (Table 1) was up-regulated in CM from LNCaP and C4-2 cells. Further exploration of each protein, on cellular level, is merited.

**Table 1:** Liquid chromatography-tandem mass spectrometry identification of proteins differentially expressed in condition media of prostate cell lines – BPH-1, LNCaP and C4-2 – resolved by two-dimensional gel electrophoresis.

Spot No.	Protein Name	Official Symbol	GenBank No.	Mw/PI*	Matched No.	Coverage	Function	Cellular Location
1, 3	Triosephosphate isomerase 1	TPI1	NM_000365	26653/6.45	19, 22	72%, 86%	Anaerobic glycolysis	Cytoplasm
1	Spondin-2	SPON2	NM_012445	35750/5.47	21	32%	Functioning as a pattern recognition receptor for microbes and an adhesion molecule for neurons.	Secreted protein
1	Thrombospondin 1, N-Terminal Domain	THBS1	NM_003246	23554/6.95	12	54%	Cell motility; cell adhesion; development; neurogenesis; blood coagulation	Secreted protein
1	Phosphoglycerate mutase 1	PGAM1	NM_002629	28786/6.67	8	29%	Anaerobic glycolysis	Cytoplasm
2	Syndecan binding protein 1	ST1	NM_005625	32397/7.05	8	28%	Cytoskeletal-membrane organization; cell adhesion; protein trafficking; the activation of transcription factors	Cytoplasm/endoplasmic reticulum/nucleus
4	Peroxiredoxin 1	PRDX1	NM_002574	22096/8.27	16	57%	Playing an antioxidant protective role in cells	Cytoplasm/nucleus
5	Nucleophosmin- isoform CRA_c	NPM1	NM_002520	13515/5.82	7	56%	Ribosome assembly; transport, control of centrosome duplication; regulation of the tumor suppressor ARF	Nucleus/cytoplasm

* : molecular weight/isoelectric point.

### Expression of SPON2 was up-regulated in CM of AR-positive PCa cell lines

**Figure 2:**
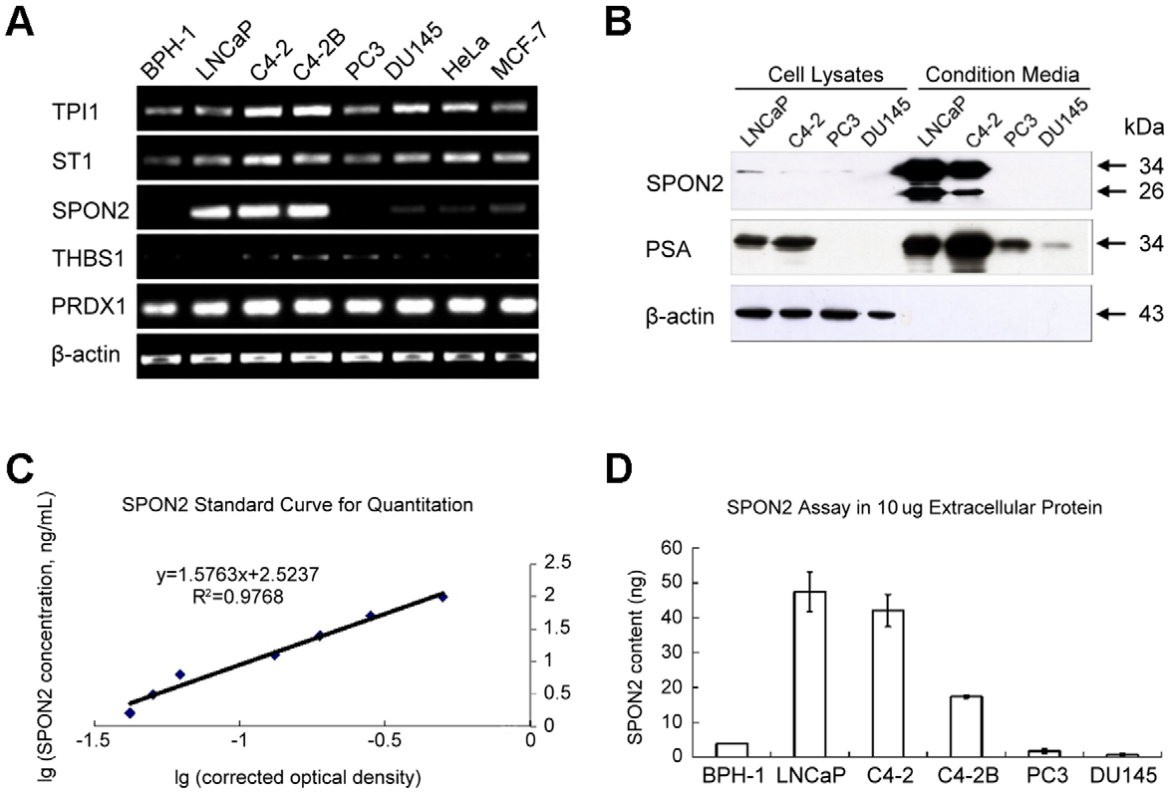
Expression of spondin-2 (SPON2) in the condition media of androgen-receptor-positive prostate cancer cell lines. (A) Semi-quantitative reverse transcription PCR analysis of five candidate molecules. (B) Western blot analysis of cell lysates and condition media protein with anti- SPON2 and anti- prostate-specific antigen (PSA) antibody. (C) Standard curve for SPON2 sandwich ELISA. (D) SPON2 quantitation in 10 µg extracellular protein in condition media of different prostate cell lines.

First, five of seven candidate molecules were identified by RT-PCR in six prostate cell lines, in cervical cancer cell line Hela, and in breast cancer cell line MCF-7. One of the candidate molecules, SPON2, in accordance with the tendency of the selected 2-DE spot, was up-regulated in CM of LNCaP and C4-2 relative to BPH-1(Figure 2A). RT-PCR results suggested that SPON2 mRNA was highly expressed in androgen-receptor-positive PCa cell lines. Western blots of cell lysates (CL) and CM protein also demonstrated that SPON2 was only positive in the CM of LNCaP and C4-2 (Figure 2B). This was consistent with the results of semi-quantitative RT-PCR. We established a method for SPON2 sandwich ELISA quantitation. By plotting the log of the corrected optical density readings (x-axis) versus the log of their corresponding SPON2 concentration (y-axis), a linear equation was fitted as Figure 2C. SPON2, in 10 µg concentrated extracellular protein in CM from different prostate cell lines, was quantitated by sandwich ELISA, as shown in Figure 2D. We found, in accordance with the results of semi-quantitative RT-PCR, that SPON2 was only expressed in the CM of AR-positive PCa cell lines LNCaP, C4-2, and C4-2B. We selected SPON2 as a candidate for histological and serological testing.

### Immunohistostaining suggested SPON2 was a potential biomarker for PCa

**Figure 3:**
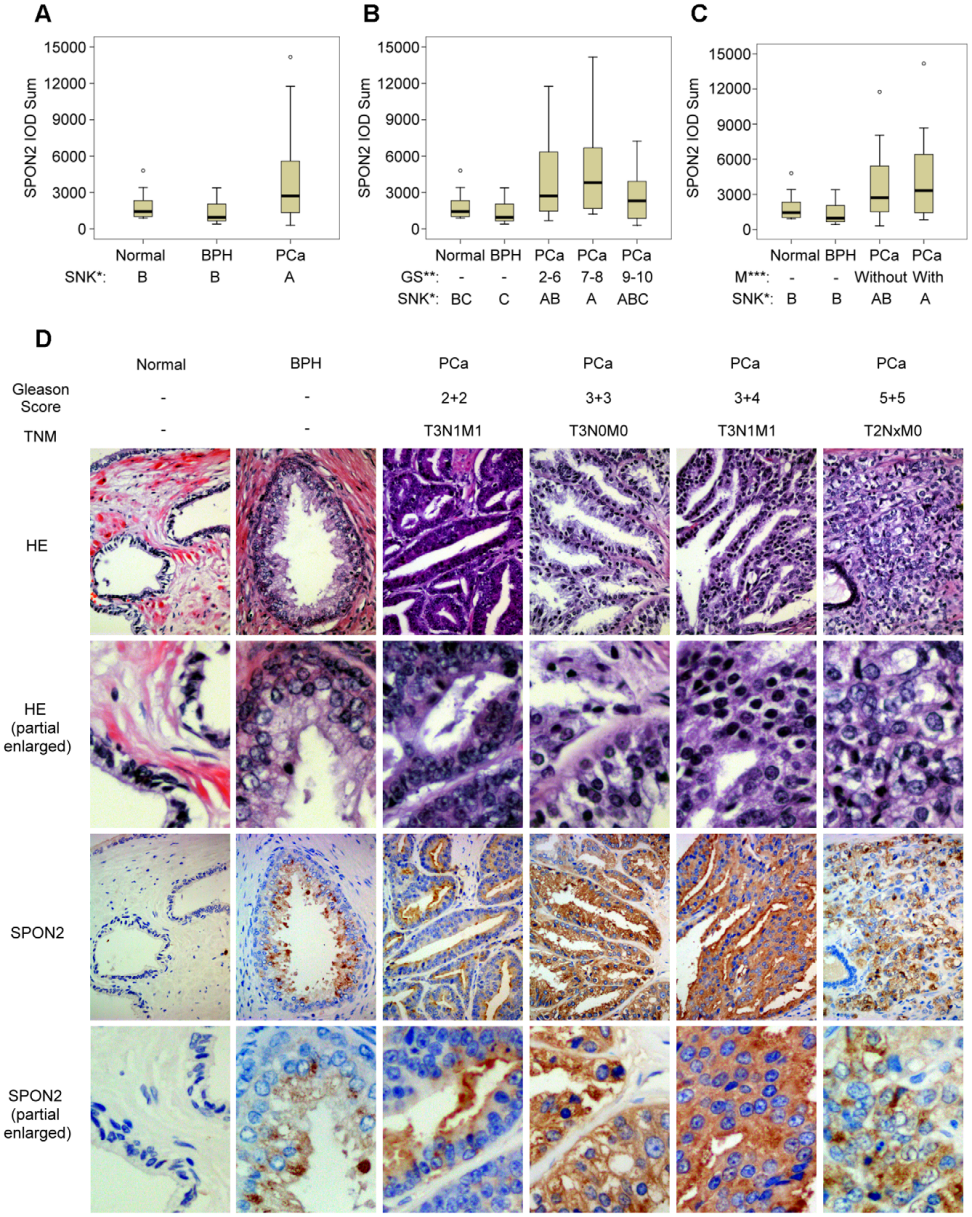
Spondin-2 (SPON2) immunohistostaining analysis of prostate tissue microarray. SPON2 integral optical density (IOD) sums of all prostate cancer patients (A), group Gleason score sum 7–8 (B) and metastasis (C) were all higher than those of both normal and benign prostatic hyperplasia (SNK* represents SNK grouping test results and groups with the same letter are not significantly different; GS** represents Gleason score sum; M*** represents metastasis status.). Same regions of same samples staining with hematoxylin and eosin (HE) and SPON2 separately were shown in (D).

Two sections from one tissue block separately stained with hematoxylin & eosin (HE) and with SPON2 are shown in Figure 3. HE staining is shown as the control. The expression of SPON2 in different cases is also shown in Figure 3A–D. The mean integrated optical density (IOD) for SPON2 protein expression (immunohistochemistry) in 73 specimens containing 10 normal prostate tissue samples, 19 BPH tissue samples, 44 PCa tissue samples with different Gleason scores, and samples of PCa tissue both with and without metastasis were calculated using Image-Pro Plus 6.0 software (Table S1).

SPON2 IOD sums were represented by median (interquartile range), e.g. M (Q_R_), and statistically tested by SNK grouping test (q test). We found that SPON2 expression was higher in PCa than in either normal or BPH tissue samples, to a statistically significant extent (Figure 3A).

The PCa specimens were divided into three groups by Gleason score sum: Gleason score sums no more than 6, Gleason score sums 7 or 8, and Gleason score sum 9 or 10. We found SPON2 expression in the group sums 7 or 8 to be higher than in either the normal or BPH tissue samples, to a statistically significant degree. Specimens that were sums no more than 6 showed significantly more expression than BPH (Figure 3B).

PCa specimens were also divided into two groups based on patients' status: specimens from patients without metastasis (M0) and specimens from patients with metastasis (M1). We found that SPON2 expression was significantly higher in group M1 than in the normal or BPH tissues (Figure 3C).

### Serum SPON2 levels were elevated in PCa patients

**Figure 4:**
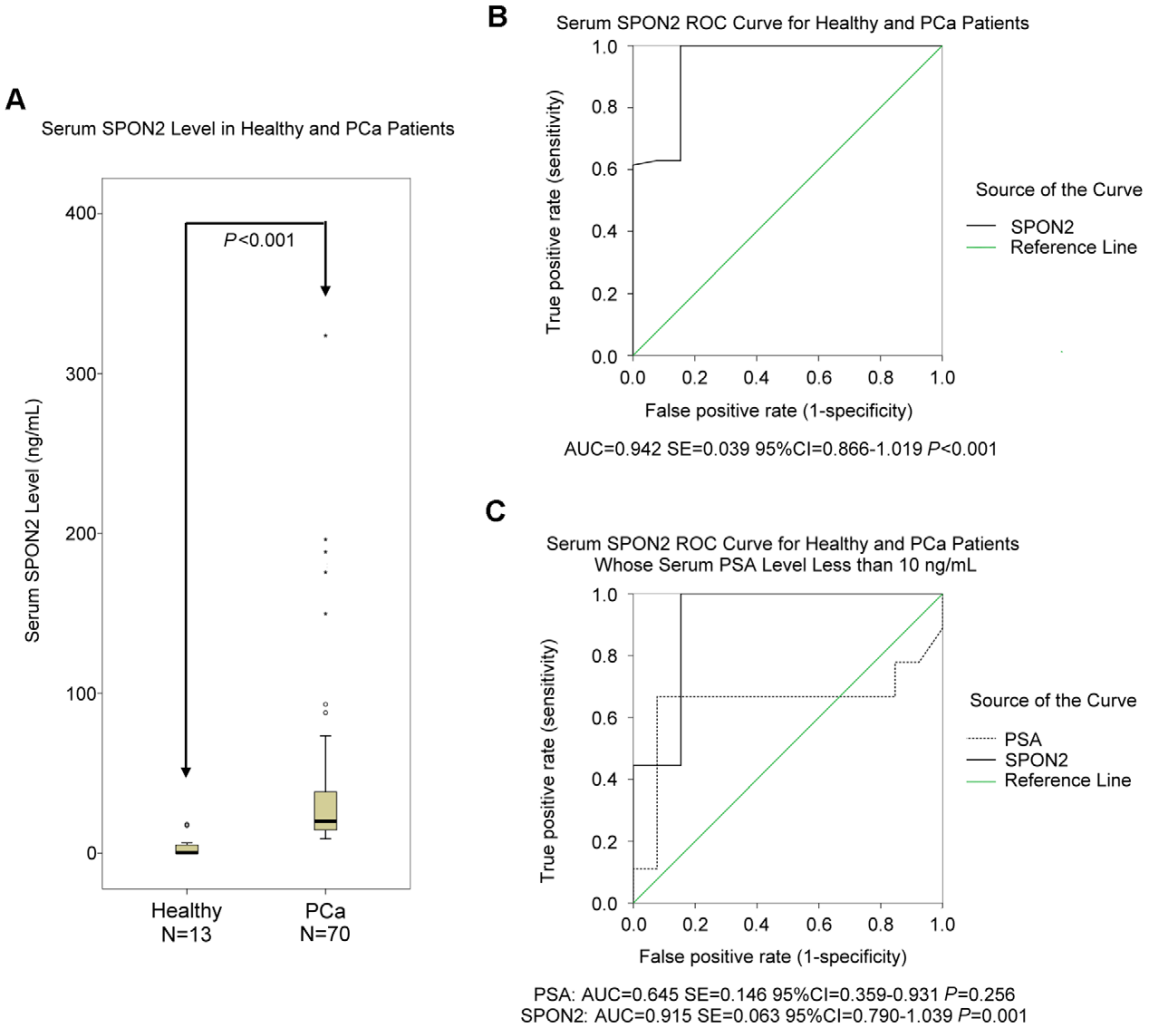
Serum concentration testing of spondin-2 (SPON2). (A) SPON2 in 70 prostate cancer patients and 13 healthy elderly men were compared. Willcoxon 2 sample testing was used to determine the significance of the differences between two groups. (B) A SPON2 receiver operator characteristic (ROC) curve for above cases is shown. (C) SPON2 and prostate-specific antigen (PSA) ROC curves for prostate cancer patients with serum PSA≤10 ng/mL and healthy elderly men are shown.

The serum SPON2 levels of 13 healthy, elderly men and 70 PCa patients (Table S2) were quantitated using SPON2 sandwich ELISA with the above-mentioned standard curve (Figure 2C). We found serum SPON2 level of PCa patients (95% confidence interval, 95%CI = 27.55–52.69) was significantly higher than that of healthy, elderly men (95%CI = 0–7.78) (*P*<0.001, Figure 4A). Sensitivity, specificity, AUC, and all cutoff values of SPON2 levels were determined using ROC analysis. SPON2 cutoff values of 3, 6, 8, 10, 15, and 20 ng/mL yielded sensitivities of 100%, 100%, 100%, 94.3%, 75.7%, and 54.3% and specificities of 69.2%, 76.9%, 84.6%, 84.6%, 84.6%, and 100%, respectively. When 8 ng/ml was chosen as the cutoff value, the sum of sensitivity (100%) and specificity (84.6%) was the highest, and the positive and negative predictive values were 97.2% and 100%, respectively (AUC = 0.942, SE = 0.039, 95% CI = 0.866–1.019, *P*<0.001).

The 70 PCa patients were divided into two groups, 9 with serum PSA level ≤0 ng/mL and 61 with serum PSA level >10 ng/mL. The serum SPON2 and PSA levels of these two groups of patients were compared to a group of healthy, elderly men (Table 2). The serum SPON2 of PCa with PSA≤10 ng/ml and >10 ng/ml were significantly higher than that of healthy controls (*P* = 0.0013 and *P*<0.001). As expected, serum PSA levels of PCa>10 ng/ml were significantly higher than those of healthy controls, while PSA≤10 ng/ml did not show any statistical significance against healthy controls (*P* = 0.2852). ROC analysis for serum SPON2 and PSA of PCa patients with PSA levels ≤10 ng/mL and healthy controls were also conducted (Figure 4C). The AUCs were 0.915 (SE = 0.063, 95%CI = 0.790–1.039, *P* = 0.001) and 0.645 (SE = 0.146, 95%CI = 0.359–0.931, *P* = 0.256), respectively. For the serum SPON2 levels of these patients and the healthy controls, a cutoff value of 8 ng/ml gave sensitivity of 100% and specificity of 84.6%. For PSA, at the recommended 4 ng/ml cutoff value, sensitivity and specificity were only 44.4% and 92.3%, respectively.

## Discussion

All the proteins secreted by viable cells constitute an organism's “secretome”. Some secreted proteins are released into the blood, so secretome research may help to reveal novel candidate serum biomarkers with potential clinical significance [10].

Gross et al. compared secretome protein profiles in medium from LNCaP cells stimulated with androgens, estrogen, and interleukin-6 and discovered that β2-microglobulin (B2M) became specifically up-regulated in the condition-medium of androgen-induced LNCaP cells [11]. Further research suggested that serum B2M levels are elevated in patients with metastatic, androgen-independent prostate cancer. Sardana G. performed a qualitative proteomic analysis of CM from the PCa cell line PC3(AR)_6_ and discovered a new PCa serum biomarker, Mac-2BP [12]. This suggests that the analysis of CM may reveal more novel, valuable PCa serum biomarkers.

SPON2 (spondin-2, Mindin, DIL-1) belongs to the F-spondin family of secreted extracellular matrix proteins [13]. SPON2 is differentially expressed in cancerous and non-cancerous lung cells [14]. It is essential to the initiation of the innate immune response and represents a unique pattern-recognition molecule in the extracellular matrix for microbial pathogens [15]. Another study reported that it serves as an integrin ligand and is critical for inflammatory cell recruitment [16]. Recent research has suggested that R-spondin family members regulate the Wnt pathway by a common mechanism and that R-spondin2 is a secreted activator of Wnt/β-catenin signaling [17], [18]. Iris Simon discovered that SPON2 is up-regulated in sera of ovarian cancer patients [19]. Renate Parry's research suggested that SPON2 mRNA is expressed predominantly in the prostate (both tumor and normal samples), and at significantly lower levels in other tissues [20]. Romanuik's analysis demonstrated that SPON2 is enriched in human prostate cancer cell lines over in other human cancer cell lines [21]. Using LNCaP tumor xenograft nude mice models, SPON2 was used for antibody-based radiotherapy of prostate cancer [20]. Sardana et al analyzed the CM proteomics from the PC3, LNCaP, and 22Rv1 prostate cancer cell lines by two-dimensional chromatography and tandem mass spectrometry, obtained four candidate biomarkers including spondin-2, and discovered that its expression level in sera showed a significant difference between patients with or without prostate cancer [22]. All these results suggest SPON2 may be a new biomarker for PCa.

**Table 2:** Quantitating serum SPON2 levels in healthy and prostate cancer (PCa) patients with different levels of serum prostate-specific antigen (PSA) by sandwich ELISA (ng/ml).

Diagnostic Conclusion		Number of Cases	SPON2 M(Q_R_)*	PSA M(Q_R_)
**Healthy**		13	0.45 (5.19)	0.40 (2.30)
**PCa**	**PSA**≤**10 ng/ml**	9	16.01 (15.06)	3.94 (5.16)
	**PSA**>**10 ng/ml**	61	21.53 (27.24)	88.50 (274.78)

* : median (interquartile range).

A prostate tissue microarray was immunohistostained with anti-SPON2 antibody. We found that the SPON2 staining intensity of PCa was significantly more intense than that of healthy, elderly men and BPH patients. SPON2 staining was more intense in sample with Gleason score sums 7–8 and in samples from metastatic PCa patients.

We used a sandwich ELISA protocol to compare serum SPON2 levels of 13 healthy controls and 70 PCa patients using Willcoxon 2 sample testing and to assess the diagnostic efficiency using ROC curves. We found that the serum SPON2 level was significantly elevated in PCa patients. The area under the ROC curve was larger than 0.90, indicating accuracy. Serum SPON2 concentrations <8 ng/mL gave the best positive and negative predictive values, 97.2% and 100%, respectively, the best sensitivity (100%), and the best specificity (84.6%).

By Willcoxon 2 sample testing, we also found that serum SPON2 levels of PCa patients whose serum PSA level was at or below 10 ng/mL was significantly higher than that of healthy controls, but their serum PSA levels were not. Based on the AUCs and their 95% CI and P values, ROC curves for serum SPON2 and PSA suggested that SPON2 can differentiate PCa patients with serum PSA≤10 ng/ml from healthy, elderly controls and that PSA cannot.

Regrettably we did not collect any sera from BPH, acute prostatitis, or prostatic intraepithelial neoplasia candidate patients, partly because of patients' preferences and partly because of the difficulties of excluding concomitant PCa without biopsy. Further studies on these patients are needed, but combining the results with the above-mentioned immunohistostaining results, we believe that SPON2 shows potentials as a more PCa-specific serum biomarker than PSA.

Based on these results, we concluded that SPON2 was a new serum and histological diagnostic biomarker for prostate cancer, showing independent diagnostic value and an ability to avoid some of the problems inherent in PSA.

## Materials and Methods

### Ethics Statement

Serum samples were collected before any clinical treatment from the Cancer Hospital of the Chinese Academy of Medical Science (Table S2). All volunteers were native Chinese, xanthoderm. Seventy PCa inpatients from this hospital also volunteered. Their diagnoses were verified by biopsy, and all gave their written informed consent. Thirteen healthy, elderly men (also with written informed consent) volunteered to serve as the control group. Only men found by DRE and B-mode ultrasound to have no prostate legions were allowed to serve as controls. The protocols for collection and analysis of the samples were approved by the National Cancer Center (NCC) Ethics Committee of China in accordance with the current revision of the Helsinki Declaration. Samples were stored at −80°C until analysis. The serum PSA levels of the patients were quantitated using a Roche Elecsys automatic electrochemiluminescence immunoassay as well as its supplementary PSA electrochemiluminescence detecting kit (Roche Diagnostics, GmbH). Corresponding standard curve preparation and serum PSA quantitation strictly obeyed the operation protocol offered by Roche.

### Cell culture

Human prostate carcinoma cell lines, LNCaP, C4-2, C4-2B (also derivative cell line of LNCaP that is androgen-independent, highly tumorigenic, and metastatic [9]), PC3, and DU145, which had been used in reference [23] were obtained as gifts from Leland W. K. Chung (Cedars Sinai Medical Center, Los Angeles, CA, U.S.); cell line BPH-1 was a gift from J. Zhang (Nankai University, Tianjin, China, PR.) and had been used in reference [24]. Breast cancer cell line MCF-7 and cervical cancer cell line HeLa were purchased from ATCC and preserved in our lab. They were grown in RPMI 1640 medium (Gibco, Paisley, U.K.) supplemented with 10% fetal bovine serum.

### Protein separation by 2-DE and silver staining

Collection, assay, concentration, and purification of proteins from CM were performed as in reference [25]. Precipitated protein samples (80 ug) were loaded for 2-DE protein separation using 7 cm ReadyStrip IPG Strips (pH 3–10, linear) in strict accordance with the product manual *2-D Electrophoresis for Proteomics* (Bio-Rad), Each experiment was repeated independently three times. The gels were silver-stained as in reference [26]. Scanning was carried out with a flatbed scanner and processed with PDQuest software (Bio-Rad). Each gel was normalized by total valid spot intensity, and differentially expressed spots were defined as spots with more than a two fold difference between cell lines.

### In gel digestion, LC-MS/MS, and database querying

In gel digestion, LC-MS/MS, and database querying are routine operations in the instrument center of our institute. Protocols are listed in Text S1.

### Semi-quantitative reverse transcription PCR (RT-PCR)

Total RNA was purified using Trizol Reagent (Invitrogen Life Technologies). First-strand cDNA was generated using the First Strand Synthesis System (TOYOBO, Japan) according to the manufacturer's instructions. Primers for semi-quantitative RT-PCR are shown in Table S3.

### Western blot

Extracellular proteins in CM and holoproteins in CL were harvested and assayed as described in reference [25]. Western blot analyses were also performed as described, except we used goat anti-human SPON2 (R&D Systems), goat anti-human PSA, and rabbit anti-human β-actin (Santa Cruz Biotechnology, Inc.) antibodies [25].

### Immunohistochemical staining

A human prostate tissue microarray was purchased from Chaoying Biotechnology Co., China, and all its included subjects were native Chinese. Tissue microarray was immunohistochemically stained with goat anti-SPON2 antibody (R&D Systems, Inc., 1∶50 dilution) as recommended by Zhongshan Goldenbridge Biotechnology, Co., China.

Five randomly selected 400× visual fields per specimen were photographed and the SPON2 IOD sum of each field was calculated using Image-Pro Plus 6.0 software (Media Cybernetics, Inc.). The IOD Sum of each specimen, i.e. the arithmetic mean of IOD sums of five randomly selected fields, was reflective of SPON2 expression level in the prostate tissue. Prostate cancer tissues were graded according to the Gleason scoring and TNM staging systems. Tumor details were extracted from medical records by Chaoying Biotechnology Co. (Table S1).

### Establishment of SPON2 sandwich ELISA quantitation protocol

A 96-well polystyrene microplate was coated with goat anti-human SPON2 polyclonal antibody (R&D Systems, Inc.) at 4°C overnight and then washed three times with PBST (PBS with 0.1% tween 20 (v/v)) and blocked (blocking buffer: PBST with 1% bovine serum albumin (BSA, v/v)). SPON2 recombinant protein (Abnova, Inc.) was diluted with blocking buffer to specific concentrations: 100, 50, 25, 12.5, 6.25, 3.13, and 1.57 ng/mL. These were used as standards. Blocking buffer served as the zero standard (0 ng/mL). The blocking buffer was discarded, and each standard was added to the microplate at 100 µl per well with two repetitions. The plate was then incubated. After three washes with PBST, mouse anti-human SPON2 monoclonal antibody (Abnova, Inc.) diluted with blocking buffer was added to the microplate and incubated. After three washes with PBST, diluted corresponding secondary antibody was added and incubated. After three washes with PBST, soluble TMB single-substrate solutions were added to each well and incubated for 15 minutes. The optical density readings at 570 nm were subtracted from the readings at 450 nm. The arithmetic mean of two zero-standard wells was subtracted from that of the two repeated wells. These values served as the corrected optical density readings of their corresponding SPON2 concentrations. By plotting the log of corrected optical density readings (x-axis) versus the log of their corresponding SPON2 concentrations (y-axis), a linear equation was fitted as the standard curve for SPON2 sandwich ELISA quantitation using Microsoft Office Excel 2003.

### Serum and CM SPON2 quantitation by sandwich ELISA

Sera were 1∶2 diluted by blocking buffer for sampling. Blocking buffer was used to dilute 10 µg amounts of assayed and concentrated CM protein to a final volume of 100 µl for sampling. Blocking buffer served as the zero standard. According to the above-mentioned sandwich ELISA protocol, except what we used for sampling, we subtracted the zero standard well from each well to provide corrected optical density readings of each corresponding sample. Each log of corrected reading as x and substituted into above-mentioned standard curve, for serum samples, 3×10^y^ ng/mL was taken to be the SPON2 concentration of the corresponding serum. For CM of different cell lines, 0.1×10^y^ ng was the SPON2 content in 10 µg concentrated extracellular protein in CM.

### Statistical analysis

Willcoxon 2 sample testing was used to determine the significance of the differences between two groups of skewness distribution data, and *P*<0.05 was considered significant. For more than three distribution measurement data groups, we used SNK grouping (q testing) and Kruskal-Wallis testing. All of the above analyses were performed using the statistical software SAS 8.0 for Windows. ROC curves were built using SPSS 13.0 for Windows.

## Supporting Information

Figure S1 **LC-MS/MS identification of selected spots.** A. Mowse score of spot 1. B. Sequence coverage of peptides to SPON2 (Matched peptides shown in Bold). C. Sequence coverage of peptides to THBS-1 (Matched peptides shown in Bold). D. Sequence coverage of peptides to TPI1 (Matched peptides shown in Bold). E. Sequence coverage of peptides to PGAM1 (Matched peptides shown in Bold). F. Mowse score of spot 2. G. Sequence coverage of peptides to ST1 (Matched peptides shown in Bold). H. Mowse score of spot 3. I. Sequence coverage of peptides to TPI1 (Matched peptides shown in Bold). J. Mowse score of spot 4. K. Sequence coverage of peptides to PRDX1 (Matched peptides shown in Bold). L. Mowse score of spot 5. M. Sequence coverage of peptides to NPM1 (Matched peptides shown in Bold).
(TIF)

Table S1 **Details of human prostate tissue microarray used in this research paper including SPON2 IOD sum we calculated.**
(DOC)

Table S2 **Details of PCa patients and normal aged men used in this research paper including serum SPON2 level we calculated.**
(DOC)

Table S3 **Primers for Semi-Quantitative RT-PCR.**
(DOC)

Text S1 **Protocols for In gel digestion, LC-MS/MS and Data base querying.**
(DOC)
