# Supplementary figures and images for: Spondin-2 (SPON2), a More Prostate-Cancer-Specific Diagnostic Biomarker
Source: PLoS One. 2012 May 15;7(5):e37225. doi: 10.1371/journal.pone.0037225 (PMC3352876; doi:10.1371/journal.pone.0037225)

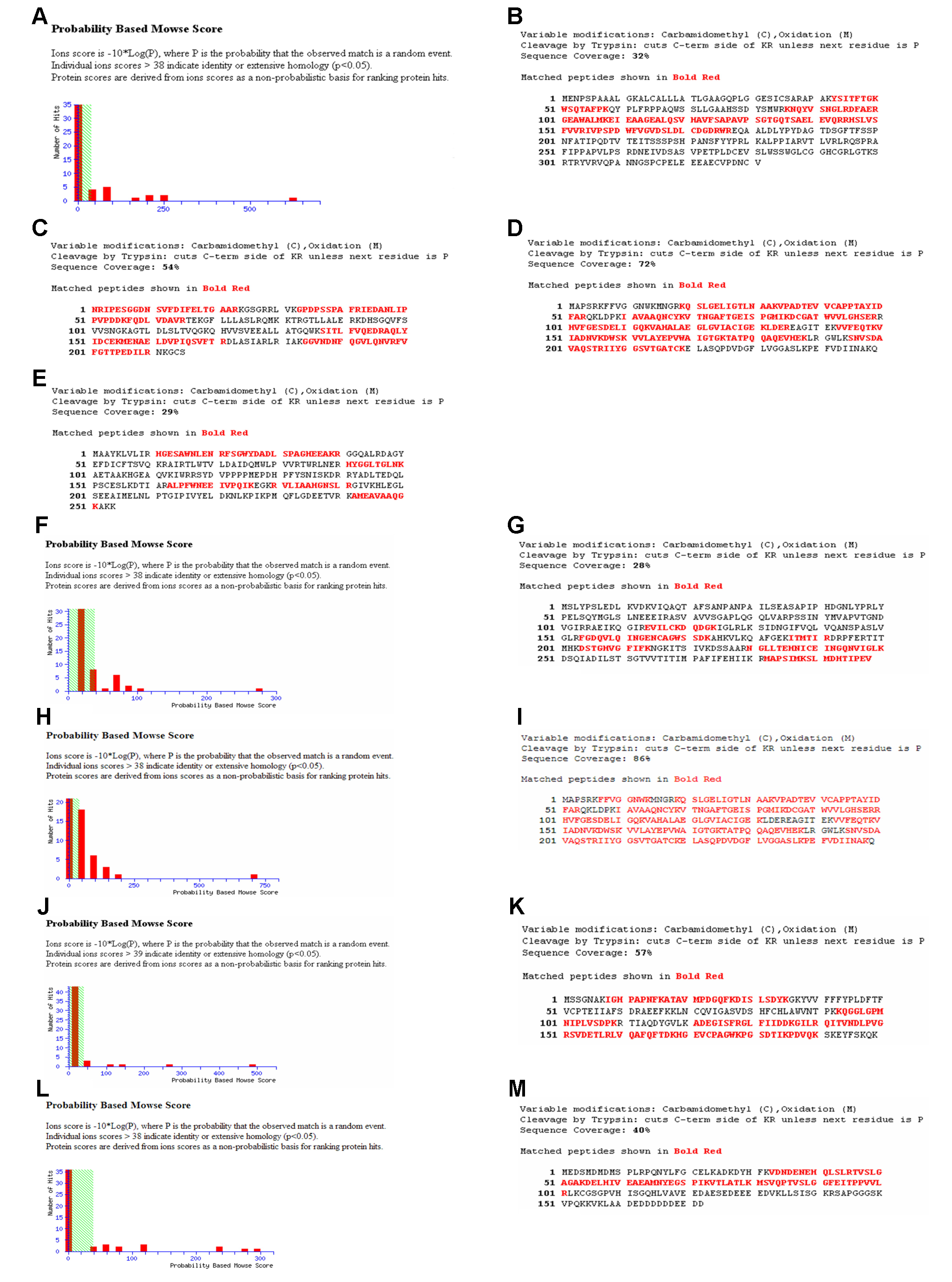

Supplement: Figure S1 — LC-MS/MS identification of selected spots. A. Mowse score of spot 1. B. Sequence coverage of peptides to SPON2 (Matched peptides shown in Bold). C. Sequence coverage of peptides to THBS-1 (Matched peptides shown in Bold). D. Sequence coverage of peptides to TPI1 (Matched peptides shown in Bold). E. Sequence coverage of peptides to PGAM1 (Matched peptides shown in Bold). F. Mowse score of spot 2. G. Sequence coverage of peptides to ST1 (Matched peptides shown in Bold). H. Mowse score of spot 3. I. Sequence coverage of peptides to TPI1 (Matched peptides shown in Bold). J. Mowse score of spot 4. K. Sequence coverage of peptides to PRDX1 (Matched peptides shown in Bold). L. Mowse score of spot 5. M. Sequence coverage of peptides to NPM1 (Matched peptides shown in Bold). (TIF) [file pone.0037225.s001.tif]
