# Supplementary material for: Spondin-2 (SPON2), a More Prostate-Cancer-Specific Diagnostic Biomarker
Source: PLoS One. 2012 May 15;7(5):e37225. doi: 10.1371/journal.pone.0037225 (PMC3352876; doi:10.1371/journal.pone.0037225)
Supplement: Table S1 — Details of human prostate tissue microarray used in this research paper including SPON2 IOD sum we calculated. (DOC) [file pone.0037225.s002.doc]

**Table S1. Details of human prostate tissue microarray used in this** research paper including SPON2 IOD sum we calculated.

| NO. | age | Pathology | Gleason score | TNM | SPON2 IOD SUM |
| --- | --- | --- | --- | --- | --- |
| 1 | 43 | Normal prostatic tissue | - | - | 9706.8 |
| 2 | 35 | Normal prostatic tissue | - | - | 10239.2 |
| 3 | 28 | Normal prostatic tissue | - | - | 11320.8 |
| 4 | 47 | Cancer adjacent normal | - | - | 48116.4 |
| 5 | 45 | Cancer adjacent normal | - | - | 15538.6 |
| 6 | 41 | Cancer adjacent normal | - | - | 9075 |
| 7 | 64 | Cancer adjacent normal | - | - | 23382.4 |
| 8 | 84 | Cancer adjacent normal | - | - | 13345.4 |
| 9 | 80 | Cancer adjacent normal | - | - | 34185 |
| 10 | 77 | Cancer adjacent normal | - | - | 20440.4 |
| 11 | 66 | Adenocarcinoma (sparse) | 1+1 | T1NxM0 | 13182.4 |
| 12 | 70 | Adenocarcinoma | 3+3 | T2aNxM0 | 70973.2 |
| 13 | 66 | Adenocarcinoma | 2+2 | T3N1M1 | 55839.2 |
| 14 | 61 | Adenocarcinoma | 3+3 | T2NxM0 | 6926.4 |
| 15 | 64 | Adenocarcinoma | 3+3 | T3NxM1 | 8302.4 |
| 16 | 76 | Adenocarcinoma | 1+2 | TxNxMx | 8030.6 |
| 17 | 69 | Adenocarcinoma | 3+3 | T2NxM0 | 16346.8 |
| 18 | 64 | Adenocarcinoma | 5+4 | T3NxM1 | 26517.6 |
| 19 | 69 | Adenocarcinoma | 3+5 | T2NxM0 | 66382.4 |
| 20 | 70 | Adenocarcinoma | 3+4 | T2NxM0 | 52544.4 |
| 21 | 78 | Adenocarcinoma | 3+3 | T4N1M1 | 53052.6 |
| 22 | 67 | Adenocarcinoma | 2+3 | T2NxM1 | 47707.6 |
| 23 | 65 | Adenocarcinoma | 3+3 | T2NxM0 | 27398.6 |
| 24 | 20 | Adenocarcinoma | 3+3 | T3N0M0 | 117491.6 |
| 25 | 73 | Adenocarcinoma | 3+3 | T2NxM0 | 80588.6 |
| 26 | 55 | Adenocarcinoma | 2+2 | T2NxM0 | 27187.2 |
| 27 | 72 | Adenocarcinoma | 4+5 | T2NxM0 | 9648.2 |
| 28 | 62 | Adenocarcinoma | 3+4 | T3N1M1 | 141639 |
| 29 | 70 | Adenocarcinoma (sparse) | 5+5 | T2NxM0 | 32008.6 |
| 30 | 70 | Adenocarcinoma | 2+3 | TxN0M0 | 73296.8 |
| 31 | 70 | Adenocarcinoma | 5+3 | T2NxM0 | 13962.2 |
| 32 | 80 | Adenocarcinoma | 3+4 | T4N1M1 | 32982.4 |
| 33 | 51 | Adenocarcinoma | 3+5 | T2N0M0 | 16850.6 |
| 34 | 58 | Adenocarcinoma | 4+3 | T2NxM0 | 17129.6 |
| 35 | 73 | Adenocarcinoma | 5+4 | T3N1M1 | 72401.2 |
| 36 | 65 | Adenocarcinoma | 4+5 | T2N1M1 | 33608.2 |
| 37 | 64 | Adenocarcinoma | 4+5 | T2NxM0 | 44732 |
| 38 | 60 | Adenocarcinoma | 3+4 | T3N1M1 | 16464.2 |
| 39 | 70 | Adenocarcinoma | 3+3 | T3NxM0 | 20382.3 |
| 40 | 73 | Adenocarcinoma | 5+5 | T4N1M1 | 8857 |
| 41 | 82 | Adenocarcinoma | 3+5 | T2NxM0 | 66803.2 |
| 42 | 76 | Adenocarcinoma | 4+3 | T3N1M1 | 12289 |
| 43 | 70 | Adenocarcinoma | 4+3 | T2N1M1 | 86872.4 |
| 44 | 40 | Adenocarcinoma | 4+5 | T2N1M1 | 10674.8 |
| 45 | 57 | Adenocarcinoma | 2+4 | T2aNxM0 | 23557 |
| 46 | 73 | Adenocarcinoma | 3+4 | T3N1M1 | 18157.6 |
| 47 | 55 | Adenocarcinoma | 4+5 | T2NxM0 | 19625 |
| 48 | 63 | Adenocarcinoma | 4+4 | T2N1M1 | 77787.2 |
| 49 | 72 | Adenocarcinoma | 5+5 | T3N0M0 | 3122.8 |
| 50 | 60 | Adenocarcinoma | 5+5 | T2NxM0 | 3174.8 |
| 51 | 87 | Adenocarcinoma | 5+5 | T2NxM0 | 39225 |
| 52 | 75 | Adenocarcinoma | 5+5 | T3NxM0 | 55941.2 |
| 53 | 57 | Adenocarcinoma | 5+5 | T4NxM0 | 3147.8 |
| 54 | 82 | Adenocarcinoma | 4+4 | T2NxM0 | 43389.2 |
| 55 | 62 | Hyperplasia of prostate | - | - | 9753 |
| 56 | 78 | Hyperplasia of prostate | - | - | 13213.4 |
| 57 | 64 | Hyperplasia of prostate | - | - | 4175.5 |
| 58 | 70 | Hyperplasia of prostate | - | - | 7593 |
| 59 | 73 | Hyperplasia of prostate | - | - | 4824.6 |
| 60 | 74 | Hyperplasia of prostate | - | - | 5082.6 |
| 61 | 81 | Hyperplasia of prostate | - | - | 33181.4 |
| 62 | 72 | Hyperplasia of prostate | - | - | 14130 |
| 63 | 78 | Hyperplasia of prostate | - | - | 6735 |
| 64 | 66 | Hyperplasia of prostate | - | - | 23689.8 |
| 65 | 79 | Hyperplasia of prostate | - | - | 6592 |
| 66 | 65 | Hyperplasia of prostate | - | - | 33893.2 |
| 67 | 81 | Hyperplasia of prostate | - | - | 17375.6 |
| 68 | 66 | Hyperplasia of prostate | - | - | 6667.2 |
| 69 | 67 | Hyperplasia of prostate | - | - | 22763.8 |
| 70 | 72 | Hyperplasia of prostate | - | - | 9303.6 |
| 71 | 56 | Hyperplasia of prostate | - | - | 33565.4 |
| 72 | 76 | Hyperplasia of prostate | - | - | 7095.4 |
| 73 | 75 | Hyperplasia of prostate | - | - | 18294.6 |
