# Supplementary material for: Spondin-2 (SPON2), a More Prostate-Cancer-Specific Diagnostic Biomarker
Source: PLoS One. 2012 May 15;7(5):e37225. doi: 10.1371/journal.pone.0037225 (PMC3352876; doi:10.1371/journal.pone.0037225)
Supplement: Table S2 — Details of PCa patients and normal aged men used in this research paper including serum SPON2 level we calculated. (DOC) [file pone.0037225.s003.doc]

**Table S2. Details of PCa patients and normal aged men used in this research paper including serum SPON2 level we calculated.**

| **Gender** | **Admission Number** | **Diagnose** | **Age** | **PSA ng/ml** | **Gleason Score** | **TNM Staging** | **Bone scan** | **SPON2 ng/ml** |
| --- | --- | --- | --- | --- | --- | --- | --- | --- |
| Male | 805551 | PCa | 66 | 18.31 | 3＋4 | T2aN0M0 | Negative | **13.97** |
| Male | 812714 | PCa | 80 | 58.8 | 2＋3 | T4N2M1b | Positive | **19.63** |
| Male | 524258 | PCa | 65 | 58.8 | 2＋3 | T3N1M1b | Negative | **323.9** |
| Male | 814829 | PCa | 66 | 3.94 | 5＋5 | T4N0M0 | Negative | **26.79** |
| Male | 808200 | PCa | 65 | 11.6 | 3＋2 | T2N0M0 | Negative | **25.95** |
| Male | 817536 | PCa | 61 | 35.91 | 3＋4 | T3N0M0 | Negative | **59.37** |
| Male | 819095 | PCa | 67 | 72 | 3＋3 | T3cN0M0 | Negative | **93.1** |
| Male | 799335 | PCa | 76 | 5.41 | 4＋5 | T3cN0M0 | Negative | **22.71** |
| Male | 822071 | PCa | 63 | 309 | 4＋4 | T3cNxM1 | Positive | **12.67** |
| Male | 823119 | PCa | 79 | 16.74 | 5＋4 | T3cN0M0 | Negative | **12.99** |
| Male | 824556 | PCa | 61 | 2148 | 3＋4 | T2N0M1b | Positive | **11.11** |
| Male | 826174 | PCa | 84 | 61.12 | 3+3 | T2N0M0 | Negative | **59.93** |
| Male | 764756 | PCa | 74 | 27.48 | 3＋3 | T1N0M0 | Negative | **25.54** |
| Male | 368671 | PCa | 76 | 34.22 | 3＋4 | T3N0M0 | Negative | **18.15** |
| Male | 830125 | PCa | 60 | 168.8 | 4＋5 | T3N1M0 | Negative | **46.96** |
| Male | 856738 | PCa | 65 | 638.1 | 4＋3 | T3N1M1b | Positive | **18.88** |
| Male | 857180 | PCa | 62 | 43.8 | 4＋4 | T3bN0M0 | Negative | **12.04** |
| Male | 858963 | PCa | 60 | 168 | 4＋3 | T2N0M0 | Negative | **149.8** |
| Male | 857477 | PCa | 58 | 29.27 | 3＋3 | T2N1M1 | Negative | **87.85** |
| Male | 850630 | PCa | 61 | 32 | 4＋5 | T3N1M0 | Negative | **27.63** |
| Male | 856240 | PCa | 62 | 948.3 | 4＋3 | T3N1M1b | Positive | **18.88** |
| Male | 851390 | PCa | 67 | 19.63 | 5＋3 | T4N1M1b | Positive | **16.71** |
| Male | 721187 | PCa | 65 | 56.04 | 5＋4 | T2bN0M0 | Negative | **23.11** |
| Male | 862188 | PCa | 77 | 12.61 | 2＋2 | T3aN0M0 | Negative | **21.92** |
| Male | 778112 | PCa | 80 | 453 | 5＋3 | T3N1M1b | Positive | **33.81** |
| Male | 862564 | PCa | 67 | 9.9 | 4＋3 | T3N0M0 | Negative | **12.35** |
| Male | 812073 | PCa | 68 | 14.65 | 4＋3 | T3bN0M0 | Negative | **16.01** |
| Male | 837104 | PCa | 76 | 1862 | 5＋4 | T3N1M1b | Positive | **20** |
| Male | 852160 | PCa | 73 | 3.52 | 4＋3 | T3N0M0 | Negative | **11.42** |
| Male | 853442 | PCa | 73 | 93.42 | 3＋4 | T2N0M0 | Negative | **16.01** |
| Male | 724980 | PCa | 60 | 100 | 4＋5 | T3N0M0 | Negative | **40.44** |
| Male | 845889 | PCa | 77 | 88.5 | 3＋4 | T3N0M0 | Negative | **175.8** |
| Male | 860336 | PCa | 65 | 110 | 4＋3 | T3N0M0 | Negative | **15.32** |
| Male | 712083 | PCa | 68 | 102 | 2＋3 | T2N0M0 | Negative | **9.92** |
| Male | 621340 | PCa | 76 | 4.47 | not known | T2N0M0 | Negative | **16.01** |
| Male | 861915 | PCa | 74 | 49.45 | 4＋4 | T4N1M1b | Positive | **36.13** |
| Male | 862409 | PCa | 69 | 541.1 | 4＋3 | T4N1M1b | Positive | **9.34** |
| Male | 862708 | PCa | 52 | 164 | 4＋3 | T4N2M10 | Negative | **18.88** |
| Male | 861317 | PCa | 81 | 22.8 | 5＋5 | T4N0M0 | Negative | **26.79** |
| Male | 721237 | PCa | 70 | 0.25 | 4＋3 | T3N0M1b | Positive | **39.46** |
| Male | 751008 | PCa | 69 | 57 | 4＋3 | T3N0M0 | Negative | **70.99** |
| Male | 861432 | PCa | 78 | 132 | 4＋3 | T2N0M1b | Positive | **12.67** |
| Male | 710340 | PCa | 72 | 37.4 | 4＋3 | T3aN0M0 | Negative | **16.71** |
| Male | 807922 | PCa | 50 | 720.5 | 4＋3 | T4N0M1b | Positive | **25.95** |
| Male | 833003 | PCa | 74 | 769.7 | 3＋4 | T3N1M1b | Positive | **15.66** |
| Male | 777744 | PCa | 64 | 130.6 | 3＋4 | T3bN2M0 | Negative | **15.32** |
| Male | 818637 | PCa | 72 | 335.5 | 4＋4 | T3bN2M0 | Negative | **22.31** |
| Male | 859885 | PCa | 57 | 1514 | 3＋4 | T4N2M1b | Positive | **73.4** |
| Male | 865914 | PCa | 48 | 1565 | 3＋4 | T4N2M1b | Positive | **188.5** |
| Male | 808988 | PCa | 57 | 6.38 | 3＋4 | T2N1M0 | Negative | **9.63** |
| Male | 826226 | PCa | 79 | 46.7 | 3＋4 | T2N0M0 | Negative | **20.38** |
| Male | 866569 | PCa | 62 | 45.2 | 5＋4 | T3N0M0 | Negative | **9.05** |
| Male | 852014 | PCa | 71 | 132 | 3＋3 | T3N0M1b | Positive | **21.53** |
| Male | 750025 | PCa | 64 | 21.57 | 1＋3 | T2N0M0 | Negative | **14.64** |
| Male | 848771 | PCa | 70 | 42.73 | 4＋4 | T3N0M1b | Positive | **42.9** |
| Male | 826274 | PCa | 65 | 100 | 4＋3 | T3N1M1b | Negative | **50.1** |
| Male | 860880 | PCa | 71 | 15.45 | 4＋5 | T2cN0M0 | Negative | **25.13** |
| Male | 846165 | PCa | 69 | 0.08 | 5＋5 | T2cN0M0 | Negative | **31.11** |
| Male | 868868 | PCa | 69 | 1524 | 4＋4 | T2cN0M0 | Negative | **196.3** |
| Male | 661478 | PCa | 76 | 0.01 | 4＋4 | T3N0M0 | Negative | **11.72** |
| Male | 851704 | PCa | 78 | 27.43 | 4＋5 | T3N1M1b | Positive | **13.97** |
| Male | 868250 | PCa | 65 | 10.93 | 4＋4 | T3N0M0 | Negative | **14.64** |
| Male | 845257 | PCa | 50 | 230 | 4＋5 | T2N1M1b | Positive | **16.71** |
| Male | 867743 | PCa | 61 | 306.7 | 4＋4 | T3N1M1b | Positive | **59.37** |
| Male | 862472 | PCa | 65 | 17.28 | 4＋4 | T3N1M1b | Positive | **20** |
| Male | 840977 | PCa | 70 | 5864 | 5＋5 | T4N1M1b | Positive | **14.3** |
| Male | 836906 | PCa | 51 | 2148 | 3＋4 | T4N1M1b | Positive | **18.51** |
| Male | 732849 | PCa | 71 | 37.14 | 4＋1 | T2N0M0 | Negative | **37.54** |
| Male | 813812 | PCa | 59 | 100 | 4＋3 | T2N0M1b | Positive | **56.58** |
| Male | 856800 | PCa | 68 | 1278 | 4＋3 | T2bN0M1b | Positive | **35.66** |
|  |  |  |  |  |  |  |  |  |
| Male | － | Normal | 58 | 0.64 | ― | － | － | **0.36** |
| Male | － | Normal | 71 | 2.81 | － | － | － | **18.15** |
| Male | － | Normal | 56 | 2.57 | － | － | － | **0** |
| Male | － | Normal | 79 | 1.03 | － | － | － | **17.42** |
| Male | － | Normal | 68 | 0.21 | － | － | － | **6.64** |
| Male | － | Normal | 67 | 0.27 | － | － | － | **0.2** |
| Male | － | Normal | 76 | 0.32 | － | － | － | **0.55** |
| Male | － | Normal | 67 | 0.29 | － | － | － | **0.45** |
| Male | － | Normal | 57 | 0.08 | － | － | － | **0** |
| Male | － | Normal | 77 | 3.3 | － | － | － | **0** |
| Male | － | Normal | 71 | 0.4 | － | － | － | **0** |
| Male | － | Normal | 53 | 8.5 | － | － | － | **5.19** |
| Male | － | Normal | 66 | 0.27 | － | － | － | **0.65** |
