# Supplementary material for: Spondin-2 (SPON2), a More Prostate-Cancer-Specific Diagnostic Biomarker
Source: PLoS One. 2012 May 15;7(5):e37225. doi: 10.1371/journal.pone.0037225 (PMC3352876; doi:10.1371/journal.pone.0037225)
Supplement: Table S3 — Primers for Semi-Quantitative RT-PCR. (DOC) [file pone.0037225.s004.doc]

**Table S3. Primers for Semi-Quantitative RT-PCR**

| **Official**  **Symbol of target gene** | **Genebank No.** | **Amplified Fragment Length(bp)** | **Annealing Temperature (℃)** | **Primer Name** | **Sequence (from 5’ to 3’)** |
| --- | --- | --- | --- | --- | --- |
| TPI1 | NM_000365 | 233 | 55 | TPI1 forward | GGCATCACTGAGAAGGTTGTTTTC |
|  |  |  |  | TPI1 reverse | CCAGTCACAGAGCCTCCATAAATG |
| SPON2 | NM_012445 | 103 | 55 | SPON2 forward | CGGCCAAATACAGCATCACC |
|  |  |  |  | SPON2 reverse | CCCAGCAGCGAAGACCACT |
| THBS1 | NM_003246 | 279 | 55 | THBS1 forward | GAGGCAGATGAAGAAGACCCG |
|  |  |  |  | THBS1 reverse | GGTGAAGACGCTTTGGATGG |
| PGAM1 | NM_002629 | 109 | 55 | PGAM1 forward | GCATGGAACCTGGAGAACCG |
|  |  |  |  | PGAM1 reverse | CATAGCCAGCATCTCGTAGCG |
| ST1 | NM_005625 | 100 | 55 | ST1 foward | TTCTCCAGCCTCATTGGTTGG |
|  |  |  |  | ST1 reverse | GCCTGTTTGAGCACCTTGTG |
| PRDX1 | NM_002574 | 101 | 55 | PRDX1 forward | CAGCTGTTATGCCAGATGGTC |
|  |  |  |  | PRDX1 reverse | CACAAAGGTGAAGTCAAGAGGGT |
| NPM1 | NM_002520 | 162 | 55 | NPM1 forward | GAACGGTCAGTTTAGGGGCTG |
|  |  |  |  | NPM1 reverse | CTTAAGACCACTGGTGGTGTTATTTC |
| β-actin | NM_001101 | 416 | 55 | β-actin forward | GAGCTACGAGCTGCCTGACG |
|  |  |  |  | β-actin reverse | CCTAGAAGCATTTGCGGTGG |
