# Supplementary material for: Spondin-2 (SPON2), a More Prostate-Cancer-Specific Diagnostic Biomarker
Source: PLoS One. 2012 May 15;7(5):e37225. doi: 10.1371/journal.pone.0037225 (PMC3352876; doi:10.1371/journal.pone.0037225)
Supplement: Text S1 — Protocols for In gel digestion, LC-MS/MS and Data base querying. (DOC) [file pone.0037225.s005.doc]

**Text S1. Protocols for In gel digestion, LC-MS/MS and Data base querying**

**In gel digestion**

Selected silver stained spot was carefully dug out, destained, and then vacuum-dried for dehydration. After reduction, the sample was washed for two times, and then placed for 15 min with methyl cyanides and vacuum-dried for 15 min. 100μL ammonium bicarbonate (m/v) solution containing 55 mmol/L iodoacetamide added, the sample was alkylated in dark room at room temperature. The sample was washed, then placed and vacuum-dried as above again. Appropriate amount of tenside and trypsin solution were added, and then the sample was placed at 4℃ for complete absorption. Appropriate amount of ammonium bicarbonate buffer added, the sample was incubated at 37℃ overnight. 20μL 5% trifluoroacetic acid (v/v) was added and the sample was incubated at 37℃ for extraction, and then the supernate was pipetted out. 20μL 50% acrylonitrile (v/v) / 2. 5% trifluoroacetic acid (v/v) was added and then the above extraction step was repeated. Two supernates were then combined and dried by vacuum concentration.

**LC-MS/MS**

Liquid phase condition: nanoACQUITY UPLC high efficiency liquid chromatography equipped with autosampler was used for liquid phase condition. The enriching column is Symmetry C18,180μm×20mm,5μm; the particle analytical column is ethane-bonded super high efficiency liquid chromatography hetero-particle column BEH C18，75μm×250mm，1.7μm; the column temperature is 35℃; the flow rate is 200nL/min; mobile phase A is aqueous solution containing 0.1%formic(v/v); mobile phase B is methyl cyanides containing 0.1%formic (v/v); the gradient is 1% to 40% phase B for 65 min, 40% to 85% phase B for 5 min, staying in 80% phase B for 10 min, and equilibrating for 20 min to starting 1% phase B.

Mass spectrum condition: Synapt High Definition Mass Spectrometry (Waters Corp., www.waters.com) was used. The ionization method is Nanospray; the data acquisition mode is DDA and the two strongest ions of each scan were analyzed by MS/MS; the capillary voltage is 2500V and the countersunk voltage is 35V; the source temperature is 90℃; the collection scopes are 350-1600 for MS and 50-2000 for MS/MS.

**Data base querying**

Mascot search engine ([http://www.matrixscience.com](http://www.matrixscience.com/)) was used for data base querying by MS/MS ion mode*.* Data acquisition condition: Trypsin cleavage was selected; oxidation of methionine and iodoacetamide alkylation modification of cysteine was considered in the process. The number of maximal missed cleavages is set to 1. The mass tolerance for MS and MS/MS are both ±0.2 Da.
